# Nationwide multicentre study of Nanopore long-read sequencing for 16S rRNA-species identification

**DOI:** 10.1007/s10096-025-05158-w

**Published:** 2025-05-10

**Authors:** Sofia Brunet, Anna Grankvist, Daniel Jaen-Luchoro, Maria Bergdahl, Jean-Luc Tison, Annica Wester, Karin Elfving, Jule Brandenburg, Karolina Gullsby, Christoffer Lindsten, Lars-Ola Arvidsson, Helena Larsson, Hinnerk Eilers, Anna Söderlund Strand, Mimi Lannefors, Johanna Keskitalo, Felicia Rylander, Jenny Welander, Malin Bergman Jungestrom, Miriam Geörg, Rene Kaden, Ida Karlsson, Anna-Malin Linde, Sara Mernelius, Linda Berglind, Lars Feuk, Susanne Kerje, Linda Karlsson, Andreas Sjödin, Lina Guerra-Blomqvist, Frans Wallin, Anna Fagerström, Martin Vondracek, Paula Mölling, Erika Tång Hallbäck

**Affiliations:** 1 Department of Infectious Diseases, Institute of Biomedicine, Sahlgrenska Academy, University of Gothenburg, Gothenburg, Sweden; 2 Dept of Clinical Microbiology, Sahlgrenska University Hospital, Gothenburg, Region Västra Götaland Sweden; 3 Department of Clinical Microbiology, Centrallasarettet, Växjö, Region Kronoberg County Sweden; 4 Department of Clinical Microbiology, Centralsjukhuset i Karlstad, Karlstad, Region Värmland County Sweden; 5 Department of Clinical Microbiology, Falu Lasarett, Region Dalarna County, Falun, Sweden; 6 Department of Clinical Microbiology, Gävle Sjukhus, Gävle, Region Gävleborg County Sweden; 7 Department of Clinical Microbiology, Hallands Sjukhus Halmstad, Halmstad, Region Halland County Sweden; 8 Department of Clinical Microbiology, Länssjukhuset Kalmar, Kalmar, Region Kalmar County Sweden; 9 Department of Clinical Microbiology, Norrlands Universitetssjukhus, Umeå, Region Västerbotten County Sweden; 10 Department of Clinical Microbiology, Skåne University Hospital, Lund, Region Skåne County Sweden; 11 Center for Molecular Diagnostics, Skåne University Hospital, Lund, Region Skåne County Sweden; 12 Department of Clinical Microbiology, Sunderby Sjukhus, Luleå, Region Norrbotten County Sweden; 13 Department of Clinical Microbiology, Sundsvalls Sjukhus, Sundsvall, Region Västernorrland County Sweden; 14 Department of Clinical Microbiology, and Department of Biomedical and Clinical Sciences, Linköping University, Linköping, Sweden; 15 Department of Laboratory Medicine, Västmanland Hospital, Västerås, Region Västmanland County Sweden; 16 Department of Medical Sciences, Clinical Microbiology, Uppsala University, 751 85 Uppsala, Sweden; 17 Clinical Genomics Uppsala, Science for Life Laboratory, Uppsala University, 751 85 Uppsala, Sweden; 18 Department of Immunology, Genetics and Pathology, Uppsala University, 751 85 Uppsala, Sweden; 19 Department of Microbiology, Public Health Agency of Sweden, Solna, Sweden; 20 Laboratory Medicine, Jönköping and Department of Biomedical and Clinical Sciences, Linköping University, Linköping, Region Jönköping County Sweden; 21 National Genomics Infrastructure, Uppsala University, Uppsala, Sweden; 22 Swedish Defence Research Agency - FOI, Umeå, Sweden; 23 Department of Clinical Microbiology, Karolinska University Hospital and Karolinska Institute, Region Stockholm County, Stockholm, Sweden; 24 Department of Laboratory Medicine, Faculty of Medicine and Health, Örebro University, Örebro, Sweden; 25 Clinical Genomics, Faculty of Medicine and Health, Örebro University, Örebro, Sweden

**Keywords:** Nanopore sequencing, 16S rRNA, Bacterial identification, EMU, Genomic Medicine Sweden

## Abstract

**Purpose:**

Recent improvements in Nanopore sequencing chemistry has made it a promising platform for long-read 16S rRNA sequencing. This study evaluated its clinical utility in a nationwide collaboration coordinated by Genomic Medicine Sweden.

**Methods:**

Thirteen mock samples comprised of various bacterial strains and an External Quality Assessment (EQA) panel from QCMD (Quality Control for Molecular Diagnostics) were analysed by 20 microbiological laboratories across Sweden, using the recent v14 chemistry. Most laboratories generated full-length 16S rRNA sequencing libraries using an optimized protocol for the 16S Barcoding Kit 24, while two laboratories employed in-house PCR coupled with the Ligation Sequencing Kit. The commercial 16S bioinformatic pipeline from 1928 Diagnostics (1928-16S) was evaluated and compared with the open-sourced gms_16S pipeline that is based on the EMU classification tool (GMS-16S).

**Results:**

Seventeen out of 20 laboratories successfully sequenced and analysed the samples. Laboratories that used sodium acetate-containing elution buffers faced compatibility issues during library construction, resulting in reduced read count. High bacterial load samples were generally well-characterized, whereas hard-to-lyse bacteria such as Gram-positive strains were detected at lower abundance*.* The GMS-16S tool provided improved species-level identification compared to the 1928-16S pipeline, particularly for closely related taxa within the *Streptococcus* and *Staphylococcus* genera.

**Conclusion:**

Nanopore sequencing demonstrated promising potential for bacterial identification in a clinical setting. The results prompt further optimization of the protocol to improve detection of a broader range of species. This multicentre study highlights the feasibility of implementing Nanopore sequencing into clinical microbiological laboratories, for improved national precision diagnostics.

**Supplementary Information:**

The online version contains supplementary material available at 10.1007/s10096-025-05158-w.

## Introduction

The 16S rRNA gene is widely used for efficient and reliable taxonomic identification of bacteria [1–3]. Amplicon sequencing of the 1 500–1 600 base pair gene provides a rapid alternative to traditional cultivation, particularly beneficial for detection of intracellular and slow-growing bacteria from normally sterile body sites. Conventional Sanger sequencing is typically restricted to detection of a single etiological agent. Although commercial sequence analysis tools, such as Ripseq, have improved identification of up to three species from Sanger chromatograms [4], the paradigm shift of next generation sequencing (NGS) allows for massive parallel sequencing of polymicrobial genomes. The Illumina platform provides high read quality but is limited to short reads, covering merely parts of the nine variable regions (V1-V9) of the 16Sr RNA gene. In contrast, third generation sequencing techniques such as Oxford Nanopore (ONT) offer long-read sequencing, as well as support real-time rapid results [5]. Previous studies have applied ONT 16S rRNA sequencing on clinical samples [5–10]. However, challenges remain in clinical interpretation as standardized protocols to establish cut-offs for differentiation of true infective pathogens from background flora are lacking, especially in polymicrobial infections [10].

In this nationwide collaboration, managed by Genomic Medicine Sweden we evaluated the performance of 16S rRNA sequencing using the ONT technology. The aim was to assess an optimized protocol for the 16S Barcoding Kit 24 V14, as a clinical diagnostic method. By harmonizing and standardizing the workflow, from sample preparation to bioinformatic analysis, we sought to facilitate local implementation and coequal diagnostic care throughout Sweden. A mock panel of 13 samples with bacterial strains from diverse taxonomic groups was sequenced in parallel with an EQA panel from QCMD, across multiple centres. Bacterial identification to species-level was performed using two bioinformatical analysis pipelines: the commercial 16S pipeline from 1928 Diagnostics (1928-16S) and an open-source pipeline based on the EMU classification tool (GMS-16S), developed under the national GMS initiative (https://genomicmedicine.se/en/). Results were compared based on performance across the participating laboratories to assess accuracy in species identification.

## Material and methods

### Multicentre study set-up

The study involved 20 participating laboratories, including 17 out of Sweden’s 21 regions as well as the Public Health Agency of Sweden (PHAS), the Swedish Defense Research Agency (FOI) and SciLifeLab National Genomics Infrastructure (NGI). A sample panel, consisting of GMS-mock and QCMD samples, was distributed frozen from Gothenburg to all participating laboratories. Each site independently extracted DNA using their available in-house systems. An optimized 16S Nanopore sequencing protocol was distributed to the laboratories that constructed libraries and sequenced locally. Sequencing data from all centres was shared and jointly analysed using two different analysis pipelines: 1928-16S and GMS-16S. The performance of 16S Nanopore sequencing was compared across laboratories with respect to species identification accuracy.

### Construction of bacteria mock sample panel and distribution to participants

The GMS-mock samples were designed to include a diverse range of cultivable bacteria, encompassing Gram-positive, Gram-negative and hard-to-lyse bacteria, from different taxonomic classes. The panel included both monomicrobial and polymicrobial samples (Supplementary Table I). Bacterial strains were obtained from the Culture Collection University of Gothenburg (CCUG) and diluted to a broad range of colony forming units per mL (CFU/mL). To account for species-specific differences, such as variations in 16S rRNA copy number and cell lysis efficiency, dilutions were selected based on an in-house 16S rRNA PCR assay conducted at Sahlgrenska University Hospital [11, 12]. Band intensity as assessed by agarose gel electrophoresis, ranged from strong positive to weak positive bands that were equivalent to a two-fold dilution above the limit of detection of the in-house PCR system. To mimic clinical samples, the human lung cancer cell line A549 (CCL-158, ATCC) was spiked into the samples. Two negative controls were included and processed simultaneously alongside the samples, one including phosphate-buffered saline (PBS, 1x) and A549 cells, and the other only PBS. In addition to the GMS-mock samples, an independent International External Quality Assessment (EQA) panel from Quality Control for Molecular Diagnostics (QCMD) Bacterial 16S EQA Scheme (B16SrRNA23, https://www.qcmd.org/en/) was included. This panel included a range of frozen, cultured bacterial species designed to assess laboratory performance in 16S rRNA gene sequencing (Supplementary Table I). The two sets of sample panels were frozen at −20 °C and shipped on dry ice to participant laboratories.

### DNA extraction, 16S rRNA library preparation and Nanopore sequencing

**Fig. 1:**
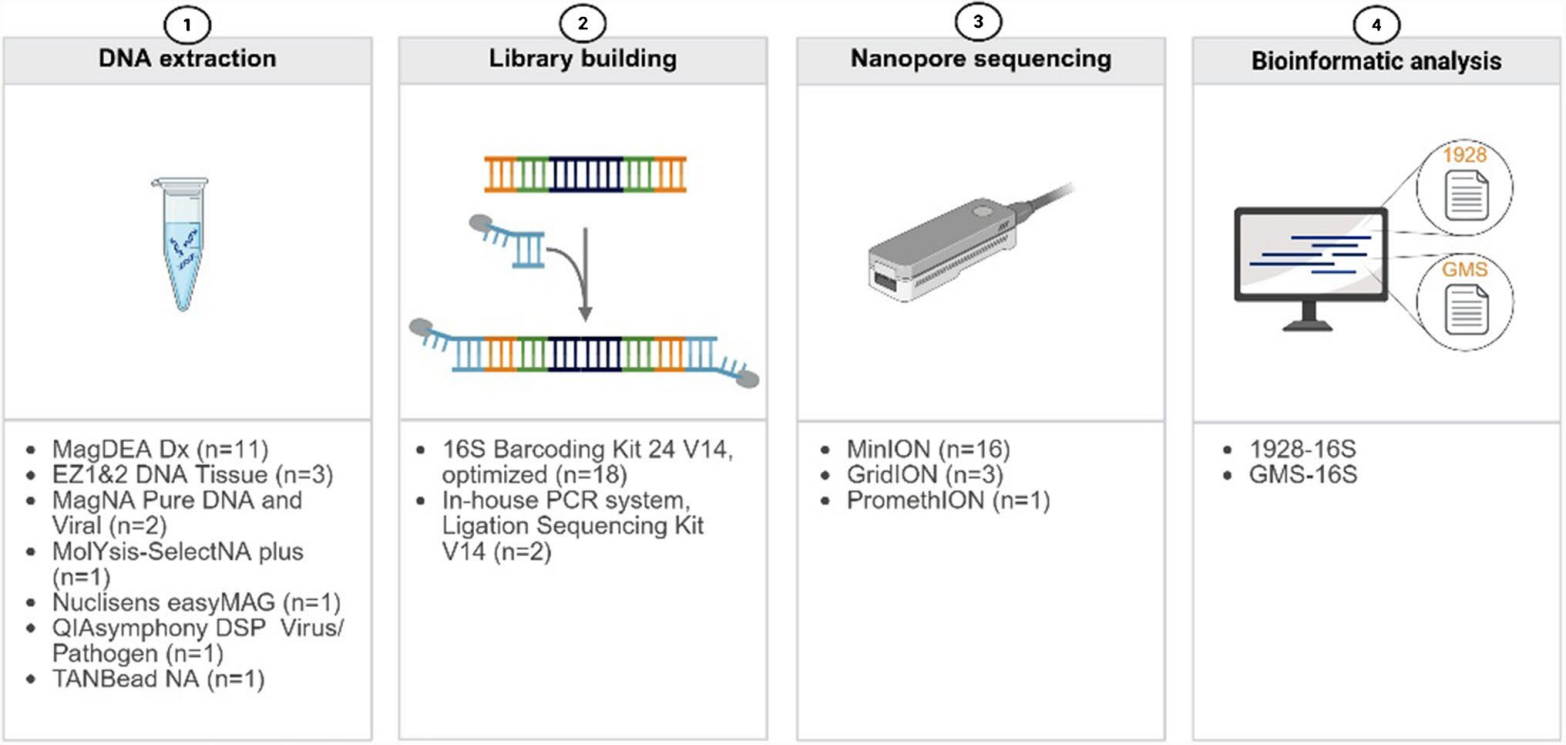
Workflow overview used by the participating laboratories. Seven different extraction methods were followed by library preparation using a modified ONT 16S Barcoding kit 24 V14 protocol (52 °C annealing, 40 cycles), or in-house PCR systems with the Ligation Sequencing V14 kit. Sequencing was conducted on various ONT devices. Species identification was performed using the commercial 1928 Diagnostics-16S pipeline and the GMS-16S pipeline (EMU classification tool). Created in BioRender. Wang, H. (2024) https://BioRender.com/i45i214

Enzymatic pretreatment, if any, and DNA extraction were performed locally at each participating laboratory, following their established protocols (Fig. 1. and Supplementary Table II). An overview of the 16S rRNA gene amplification and library preparation processes are shown in Fig. 1. Mainly, the 16S Barcoding Kit 24 V14 (SQK-16S114.24, ONT) was used for library preparation, following the manufacturer’s protocol with some modifications. The number of PCR cycles increased from 25 to 40, to account for low concentrated samples in a clinical setting. Additionally, the annealing temperature was lowered from 55 °C to 52 °C, to improve sensitivity and allow for possible mismatches in the primer binding sites. To enable applicability in a clinical setting, DNA quantification was not performed prior to amplification, instead 10 µL of DNA was directly used for library preparation. Libraries were normalized, using either *equimolar* ratios (1) or were pooled to *roughly* match concentrations (2) (Supplementary Table II). Number of pooled libraries varied from nine to 24. Sequencing was predominantly performed for 12 h using MinION, GridION or PromethION devices with FLO-MIN114 flow cells (ONT). Base calling was carried out according to Supplementary Table II, with a minimum Q score of 10.

### Bioinformatic data analysis and identification of pathogen

Locally generated data was shared and jointly analysed using two different pipelines. Initially, each laboratory uploaded the FASTQ files to the 1928 Diagnostic platform for analysis using the 1928 16S V1 V9 pipeline for amplicon data generated with Nanopore (https://www.1928diagnostics.com/). Secondly, FASTQ files were analysed using the GMS-16S pipeline (gms_16S, https://github.com/genomic-medicine-sweden/gms_16S, 1.0 dev 240416). Briefly, the pipeline involved quality control using FastQC (0.11.9), NanoPlot (1.41.0, > q12) and MultiQC [13]. Adapter trimming using Porechop_ABI is optional and was not enabled. Filtlong 0.2.1 filtered reads by length (1 200- 1 800 bp). Finally, the GMS-16S performed taxonomic profiling using EMU (v3.4.4) [14] and results were visualized using Krona 2.8 [15]. Relative abundance of reads for each sample were gathered both from the 1928 platform and calculated from the EMU output, by dividing the number of approved reads per species by the total number of reads per sample. This was done to account for potential unclassified proportions of reads that are otherwise rejected from relative abundance by default. Hits above one percentage relative abundance were further evaluated and reported.

## Results

To assess the utility of Nanopore sequencing for 16S rRNA-based clinical diagnostics, we conducted a nationwide multicentre study, analysing a mock panel across 20 microbiological laboratories. Species in samples with high bacterial load were generally identified, while detection was poor for low bacterial load samples as well as hard-to-lyse species. The GMS-16S tool improved species-level identification compared to the 1928-16S pipeline, particularly for closely related taxa within *Streptococcus* and *Staphylococcus*. Overall, both bioinformatic pipelines provided satisfactory results, with most laboratories achieving comparable genus-level identification.

### Sequencing performance

Seventeen out of 20 laboratories successfully sequenced the panels, with total number of reads per run ranging from 606 661 to 7 068 074 after quality filtering (Supplementary Fig. 1, Supplementary Table III). Although the samples were shipped on dry ice, some thawed during transit. However, this did not profoundly affect the total number of reads per run (Supplementary Fig. 1). The mean read length was 1 567 ± 63 bp for the QCMD samples and 1 484 ± 50 bp for the GMS samples, with average read quality of 16.5 ± 1.2 (QCMD samples) and 17.7 ± 1.8 (GMS samples). 77.0 ± 6.2% of reads (QCMD samples) and 80.0 ± 11.8% (GMS samples) exceeded quality score of Q15. Three laboratories (*q*, *d* and *s*) encountered compatibility issues during library preparation due to the use of sodium acetate-containing elution buffers, which resulted in fewer total reads (Supplementary Fig. 1). Additionally, laboratories which pooled libraries to *roughly* match each other (*c, b, f, y,* and *h*), as opposed to *equimolar* pooling, generally obtained lower yield for the GMS samples compared to the QCMD samples. Laboratory-*k* generated the highest number of total reads, due to extended sequencing duration (17.5 h for the QCMD samples and 24 h for the GMS samples, Supplementary Table II). Finally, one laboratory (*n*) did not successfully complete the initial sequencing run of the QCMD samples.

### 16S rRNA bacterial identification of panels

#### Monomicrobial samples

**Fig. 2:**
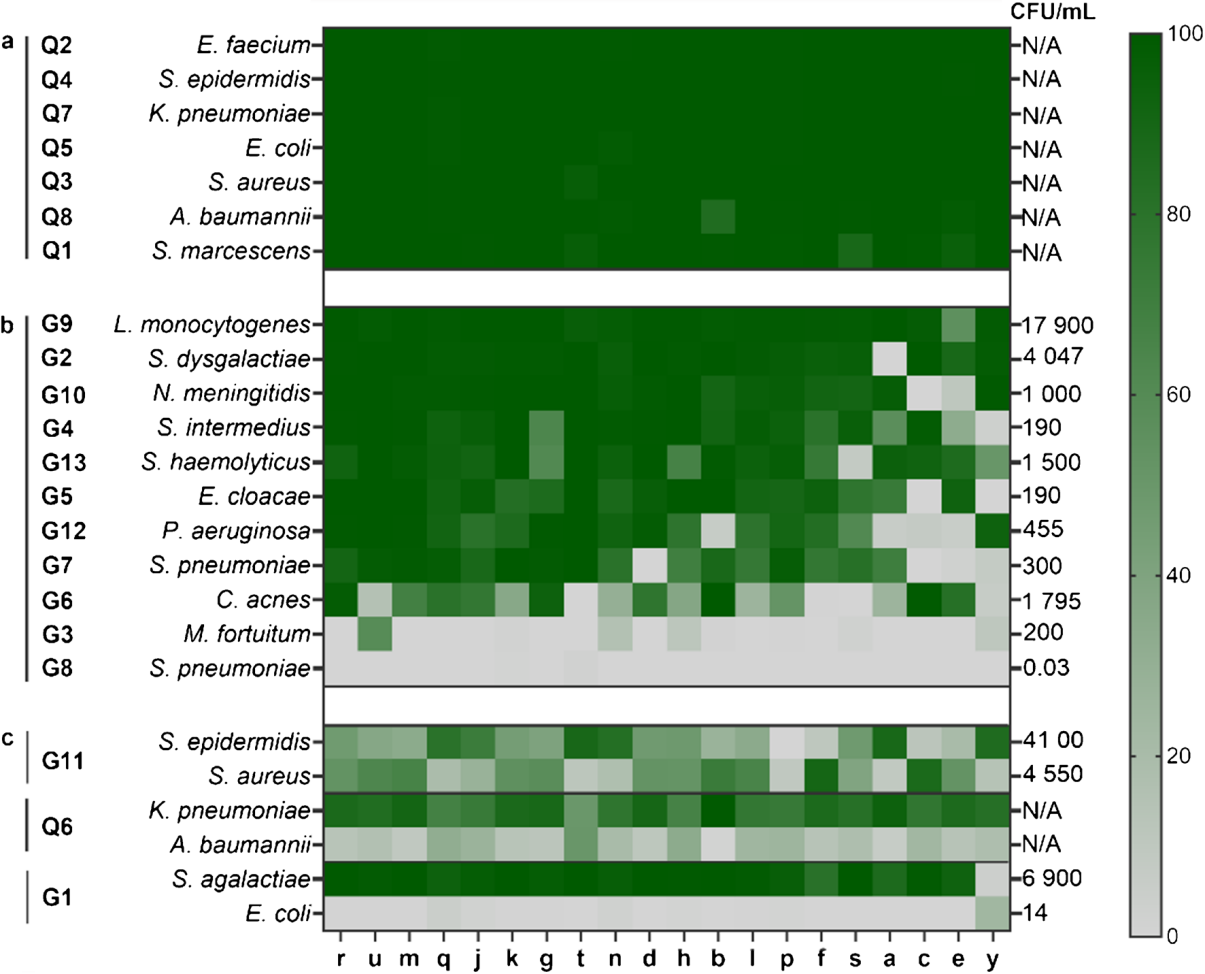
Relative abundance (%) of reads per sample for each laboratory (*r-y*) and species using the GMS-16S pipeline. **(a)** monomicrobial QCMD samples (top), **(b)** monomicrobial GMS samples (middle) **(c)** polymicrobial samples for both sample sets (bottom). Bacterial load (CFU/mL) is provided for the GMS panel, while QCMD concentrations are unknown (N/A). See Supplementary file 2 for detailed classification

All FASTQ files were shared for coordinated and harmonized analysis at Sahlgrenska University Hospital employing two separate bioinformatic pipelines, 1928-16S as well as GMS-16S. Species were reported based on relative abundance of reads in comparison to total number of reads per sample. All laboratories identified correct species in the QCMD samples with high bacterial load (Q1-Q8, Fig. 2). Detection was lower for the customized GMS samples (G1-13). The lowest concentration of bacteria detected by the majority of laboratories was 190 CFU/mL for both Gram-positive *Streptococcus pneumoniae* (G7) and Gram-negative *Enterobacter cloacae* (G5). Two monomicrobial samples with low relative abundance remained undetected by most laboratories: *S. pneumoniae* at 0.03 CFU/mL (G8) and *Mycobacterium fortuitum* at 200 CFU/mL (G3). Moreover, low levels were observed for bacteria that are challenging to lyse, such as *Cutibacterium acnes* (G6), despite its relative high concentration of 1 795 CFU/mL. Detection was nevertheless improved with enzymatic pretreatment using proteinase K, as demonstrated by laboratory-*j* in a consecutive analysis (Supplementary Table IV). Lower identification levels were occasionally observed, particularly for laboratories-*c*, *y* and *e*. This was associated with low total number of reads per sample or poor read quality, such as short reads and/or low Q-scores, leading to a high proportion of unclassified reads. Additionally, some laboratories reported reduced levels of spiked-in bacteria due to detection of an alternative species (*g*-G4 *Moraxella osloensis* 23%, *g*-G13 *M. osloensis* 22% and *a*-G12 *Acinetobacter baumannii* 91% (Supplementary File 2).

#### Polymicrobial samples with two species

The panel included three polymicrobial samples (G1, G11 and Q6). Sample G11, containing the taxonomically complex *Staphylococcus epidermidis* (4 550 CFU/mL) and *Staphylococcus aureus* (41 000 CFU/mL), showed variable detection across laboratories regardless of bacterial load. Sample Q6, with unknown bacterial load, also had inconsistent detection with relative abundance varying between *Klebsiella pneumoniae* and *A. baumannii* (99–1% to 50- 50%) across laboratories (Fig. 2). Sample G1, primarily composed of *Streptococcus agalactiae* (6 900 CFU/mL), was overall identified while detection of *Escherichia coli* (14 CFU/mL) was poor, except for laboratory-*y* where low read count affected the relative abundance. In some cases, low abundance was associated with a low total number of reads (*y*-G1, *p*-G11, *s*-G11) or a high abundance of unclassified reads due to poor read quality (*e*-G11) (Supplementary table III).

### Evaluation of two bioinformatic pipelines: 1928-16S and GMS-16S

**Fig. 3:**
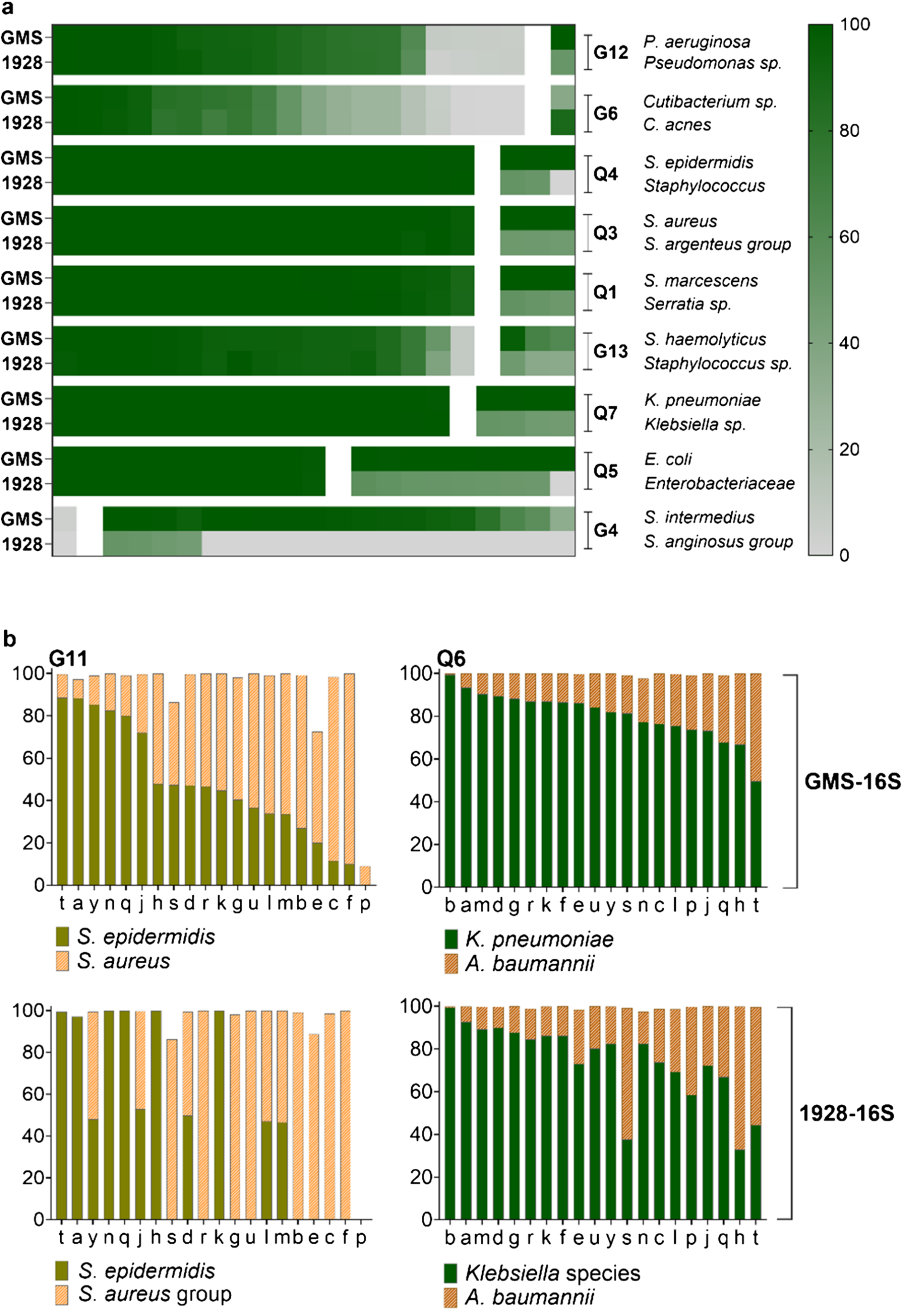
**a** Comparison of species identification between GMS-16S and 1928-16S for samples with the largest discrepancies (G12-G4). The relative abundance (%) for each laboratory is represented by a box, with similar identification on the left and differences on the right**.** See Supplementary File S2 and S3 for details. **b** Comparison of species distribution in the polymicrobial samples G11 and Q6 across the laboratories (*a*-*y*). Relative abundance (%) of reads are shown for both pipelines (GMS-16S vs 1928-16S)

The performance of two separate bioinformatic pipelines were compared: the commercial 16S pipeline developed by 1928 Diagnostics (1928-16S) and the gms_16S bioinformatics analysis pipeline that uses the EMU classification tool (GMS-16S). Overall, 1928-16S identified a higher number of species in comparison to GMS-16S (Supplementary FigS2, Supplementary file 2 and 3). However, significant differences were observed at species level, particularly for *Streptococcus* and *Staphylococcus*. GMS-16S demonstrated high accuracy of species level classification, effectively discriminating *S. intermedius* from *S. anginosus* in sample G4, as well as separating S*. aureus* from *Staphylococcus argenteus* in sample Q3 (Fig. 3a). GMS-16S also more accurately classified members of the Enterobacteriaceae family (Q7, Q5), and was able to identify *Serratia marcescens* at species level with greater precision in sample Q1 compared to 1928-16S. Conversely, 1928-16S classified a larger proportion of reads as *C. acnes* in sample G6 (laboratory *k*), whereas GMS-16S distributed the reads between *C. acnes* and the closely related *C. namnetense*. Finally, the 1928-16S pipeline reported lower levels relative abundance in four samples due to high abundance of unclassified reads (*f*-Q3 *S. aureus*, *p*-Q7 *K. pneumoniae* and *u, m*-Q1 *S. marcescens,* Supplementary file 3). In the polymicrobial sample G11 that contained two closely related species, GMS-16S successfully distributed reads between *S. epidermidis* and *S. aureus* (Fig. 3b). In contrast, 1928-16S predominantly classified reads to only one of the two species across most laboratories.

### Detection of contaminants

To assess contamination patterns, two negative controls were included in the panel. Samples with high bacterial load showed fewer classified species and possible contaminants (Supplementary Fig. 3). This was particularly evident in the QCMD panel, where only correct species was identified. Conversely, samples with low biomass (G6, G8 and G3) displayed more contaminants. Similarly, the negative control that only included PBS (NTC2) had a high number of species, as opposed to NTC1 that also included human cells to simulate the background of patient-derived material. No single contaminant was consistently detected across all laboratories (Supplementary Fig. 4, Supplementary Table VI). The most frequently identified contaminant was *C. acnes*, detected in twenty instances with a relative abundance above 10%, by nine out of twenty laboratories (Supplementary Table VII). Oher commonly detected species were those included in the highly positive QCMD samples.

## Discussion

This study evaluated the performance of Nanopore 16S sequencing by utilizing recent advancements in ONT technology in a national multicentre collaboration, involving laboratories with varying levels of experience in 16S rRNA gene sequencing and NGS techniques. Two sets of samples were analysed: a EQA panel from QCMD and a custom mock GMS panel. The primary goal was to assess the platforms´ potential as a reliable diagnostic tool for bacterial identification in clinical laboratories, using both commercial and in-house analysis protocols. Our results demonstrate that Nanopore sequencing combined with bioinformatical classification tools can accurately identify bacteria in both monomicrobial and polymicrobial samples.

Seventeen out of 20 laboratories successfully sequenced and analysed the samples provided, highlighting the user-friendliness of the Nanopore platform, even for participants with limited prior experience. Three laboratories encountered issues during library preparation due sodium acetate in the DNA eluate, which was resolved by substituting to water at elution (data not shown). Additionally, read distribution differed between sample sets based on the normalization strategy. Laboratories that pooled libraries using approximate concentrations (h, f, b, c, and y), rather than precise equimolar normalization, reported lower read counts for the GMS samples. This was likely due to read dominance by the high-bacterial load QCMD samples. These findings underscore the critical importance of accurate library quantification and normalization, particularly for clinical samples with low bacterial load.

All laboratories successfully identified correct species in the QCMD samples, demonstrating the robustness of the method. For the more challenging GMS samples, the lowest detectable concentration in monomicrobial samples was 190 CFU/mL. Samples with lower concentrations, such as 0.03 CFU/mL of *S. pneumoniae* and 14 CFU/mL of *E. coli*, were not detected. This globally aligns with previously reported detection limits of 90 CFU/mL for Gram positive *S. aureus* and 15 CFU/mL for Gram negative *E. coli* [16]. Detection of bacteria with a complex cell wall such as *C. acnes* proved challenging. Extraction of such bacteria can however be improved through enzymatic pretreatment, using for example proteinase K or mutanolysin. One laboratory in this study increased detection of *C. acnes* using proteinase K pretreatment. Although conclusions regarding efficiency of specific protocols in this study are restrained, due to differences in extraction methods and freeze-thawing of the panel, this nevertheless underscores the importance of additional pre-enzymatic treatment for comprehensive bacterial identification. This study primarily focuses on the user-friendliness of ONT sequencing for clinical microbiological laboratories, rather than establishing precise detection limits. It is hampered by the absence of replicates and possible variability across laboratories, due to the large upscaling in volume of low-concentrated samples. Future studies should address these factors, as well as expand beyond panels including solely cultivatable strains, to fully reflect the complexity of clinical samples.

A well-known limitation of the widely used 27 F primer is its underrepresentation of certain genera, such as Chlamydiales and *Borrelia* [17–19]. To mitigate this, the annealing temperature was lowered by three degrees to better tolerate primer mismatches. Our panel included *M. fortuitum* that was particularly challenging due to its low concentration combined with a low 16S copy number. Despite additional attempts with enzymatic pretreatment using proteinase K, detection remained low (11% relative abundance for laboratory-*j*) compared to a similar sample with *Streptococcus intermedius* with comparable quantity of 16S copies (G4 laboratory-*j*). We hypothesized that the persistent low level is a consequence of a mismatch to the 27 F primer. Similar false negative results were observed in later work by us and others on clinical samples containing *Chlamydophila psittaci* [5] and *Borrelia species* (data not shown). Matsuo et al. improved species coverage using modified degenerate primers for full-length 16Sr RNA sequencing on MinION [20]. Future studies will explore alternative primers to improve detection of clinically relevant species.

Bacterial identification using targeted metagenomic sequencing is highly influenced by the vast diversity of bioinformatic pipeline and classification tools. In this work, we compared a commercially available pipeline (1928-16S), which requires minimal bioinformatic expertise, to an open-source pipeline (GMS-16S). While commercial pipelines offers user-friendly interfaces, they come with higher costs for analysis and storage as well as strict requirements for patient data handling and security. In our hands, the 1928-16S pipeline identified more species overall. On the other hand, the GMS-16S pipeline demonstrated higher ability to distinguish closely related species, such as *S. intermedius* and *S. anginosus*, as well as differentiating species within the Enterobacteriaceae family. This increased specificity of GMS-16S likely derives from its integrated expectation–maximization algorithm, combined with an iterative error-correction steps that may help to overcome the high error rates typically associated with Nanopore sequencing. Moreover, the two pipelines use distinct databases with diverse genomes. GMS-16S utilizes a combination of the ribosomal RNA Operon copy number (rrnDB) and the NCBI 16S RefSeq databases. Meanwhile, the 1928-16S pipeline relies on SILVA v138.1.

Contamination remains a significant challenge for the clinical implementation of 16S rRNA NGS, as the lack of standardized protocols makes it difficult to recognize true pathogens from background contaminants. In our study, *C. acnes* was the most detected contaminant, consistent with previous experience of contamination from laboratory personnel or reagents [21, 22]. Other frequently detected contaminants were species included in the concentrated QCMD samples, suggesting cross-contamination. Despite efforts to minimize contamination, such as adhering to good laboratory practices, early-stage barcoding, and avoiding nested PCR steps, contamination remains a challenge in targeted metagenomic sequencing [23]. However, in this study, detection of contaminants was only sporadically observed and spread across different laboratories. Adequate sequencing depth is essential for sensitive and reliable detection of clinically relevant bacterial species. However, we also observed the opposite effect: at very low sequencing depths, the number of detected species appeared to increase (Supplementary Figure S3). This can elevate the risk of misidentifying background contaminants as clinically significant organisms. When the total number of reads per sample is low, the relative abundance of reads for any species becomes inflated, increasing the likelihood of false-positive findings. The development and implementation of standardized guidelines for reporting sequencing quality metrics will be crucial to minimizing this risk and ensuring accurate interpretation in clinical practice.

Various strategies have been proposed to manage contamination in NGS results. One common approach involves subtracting contaminant levels detected in negative controls from those found in samples [10]. Another general methodology uses relative abundance thresholds (typically > 1–6%). These have successfully been applied to establish background levels in monomicrobial samples [10, 16, 18, 24]. However, polymicrobial samples present a more complicated challenge, requiring additional criteria beyond read count or relative abundance [16]. Notably, thresholds-strategies assumes that contaminants are present at low abundance. However, as shown by us and others, the number of contaminants tend to increase with low biomass [25–27]. A more flexible approach uses the most abundant contaminant as a threshold for establishing positive findings [28]. Furthermore, the R-package Decontam utilizes the inverse relationship between DNA concentration after library preparation and contaminant read counts to identify and filter out contaminants [29]. However, its performance was poor when mock communities with varying compositions and concentration were used [30]. Other tools, such as microDecon and SourceTracker, can remove only a portion of the reads belonging to possible contaminant species, rather than removing all reads for that species [31, 32]. This is particularly useful when the contaminant species also is a possible pathogen. Nonetheless, these tools are most effective when the abundance of contaminants constitute less than one-third of the total abundance [27]. Moreover, the success of these tools also depend on how well the background contamination is pre-defined [27, 30]. Further evaluation is needed to determine whether such tools are time-efficient and applicable to clinical diagnostic samples. Ultimately, the best approach may involve a combination of strategies tailored to each laboratory’s specific needs, alongside careful consideration of species characteristics, such as 16S rRNA gene copy number. Finally, close collaboration with clinicians will continue to play an essential role to interpret the relevance of specific findings.

In conclusion, our work demonstrates the feasibility of Nanopore sequencing across multiple laboratories with varying methodologies. Continued development of the protocol such as extraction, primer compatibility, and bioinformatic tools will likely enhance the utility of Nanopore as a rapid and flexible tool for clinical diagnostics of 16S rRNA bacterial species identification.

## Supplementary Information

Below is the link to the electronic supplementary material.

Supplementary file1 (DOCX 552 KB)

Supplementary file2 (XLSX 188 KB)

Supplementary file3 (XLSX 337 KB)

Supplementary file4 (XLSX 220 KB)

## Data Availability

The datasets generated during the current study are available in the Sequence Read Archive (SRA) repository, SUB14925052.
