# Supplementary material for: Nationwide multicentre study of Nanopore long-read sequencing for 16S rRNA-species identification
Source: Eur J Clin Microbiol Infect Dis. 2025 May 10;44(8):1907–16. doi: 10.1007/s10096-025-05158-w (PMC12321653; doi:10.1007/s10096-025-05158-w)
Supplement: Supplementary file 1 — Supplementary file1 (DOCX 552 KB) [file 10096_2025_5158_MOESM1_ESM.docx]

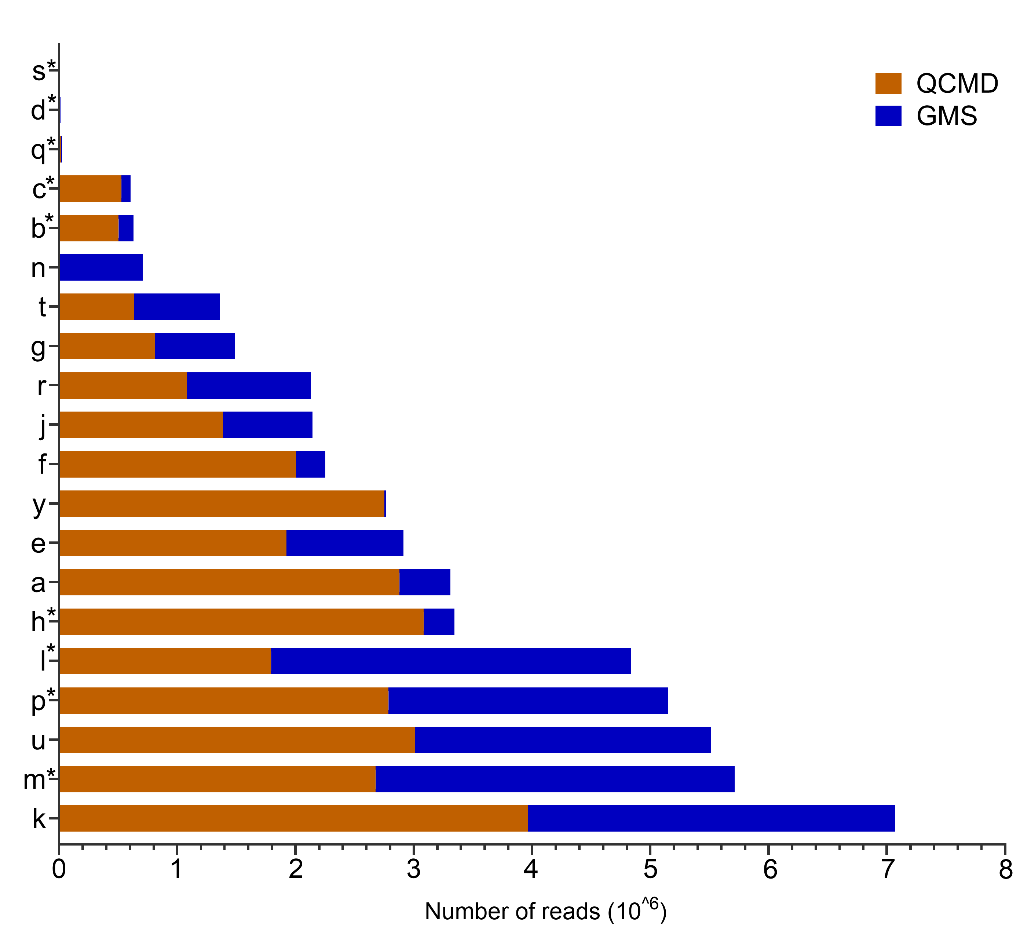


**Supplementary Figure S1**. Total number of reads per sequencing run for laboratory from GMS-16S for QCMD and GMS samples for laboratory-*s* to *k*. Asterisk (*) indicates thawed samples upon arrival at the local laboratory


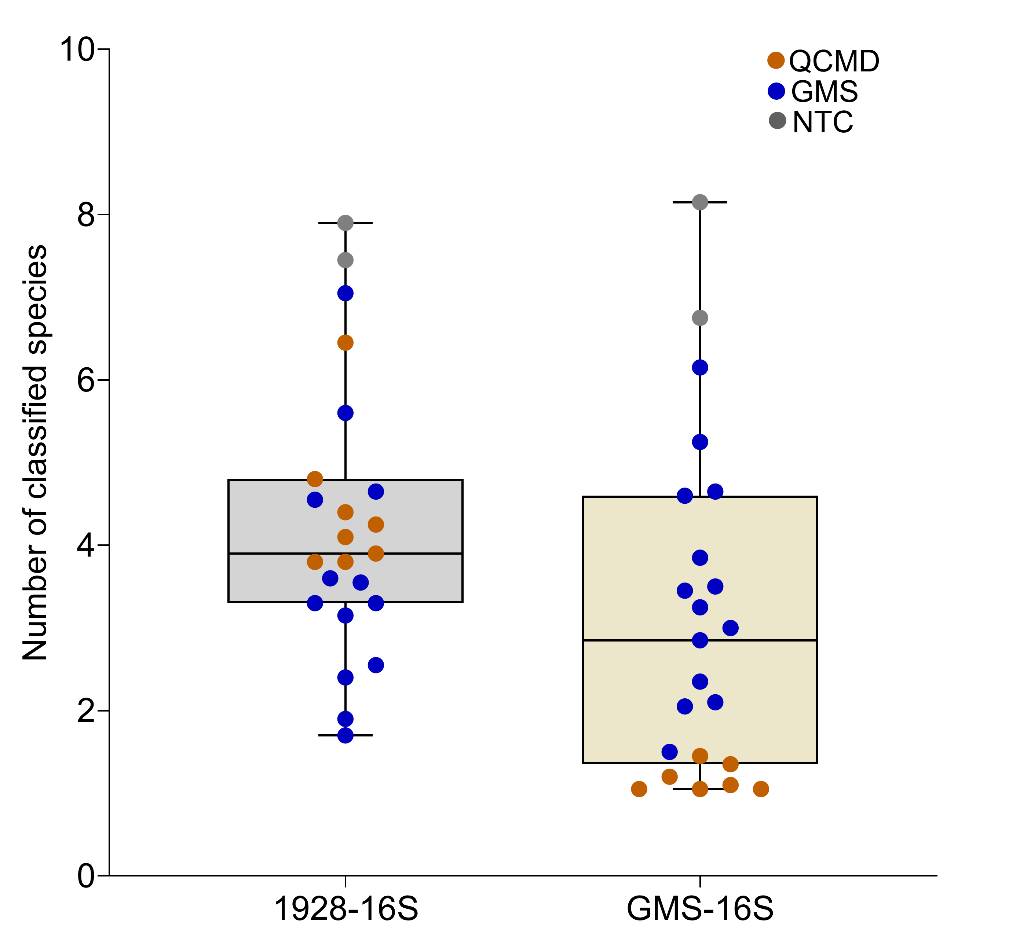


**Supplementary Figure S2**. Total number of species detected using 1928-16S or GMS-16S pipeline with a relative abundance above 1%. Each dot represents the mean of all samples (Q1-Q8, G1-G13) and negative controls with the standard deviation indicated with an error bar

**
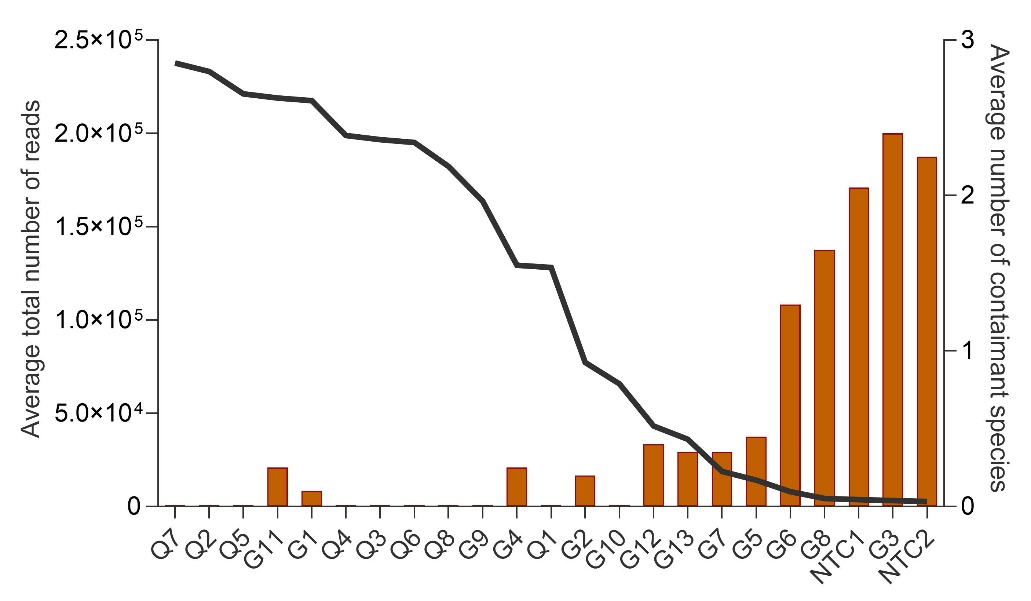
**

**Supplementary Figure S3.** Number of contaminant-classified species increases for low bacterial load samples (G3, G8), bacteria that are difficult to lyse (G6) and negative controls with low biomass (NTC2 without human cells compared to NTC1 with human cells). The bar plots represent the number of contaminant-classified species, while the line indicates the trend in relation to total read count. A higher number of contaminants is observed in samples with fewer sequencing reads. Data is based on relative abundance above 10 % obtained using the GMS-16S pipeline


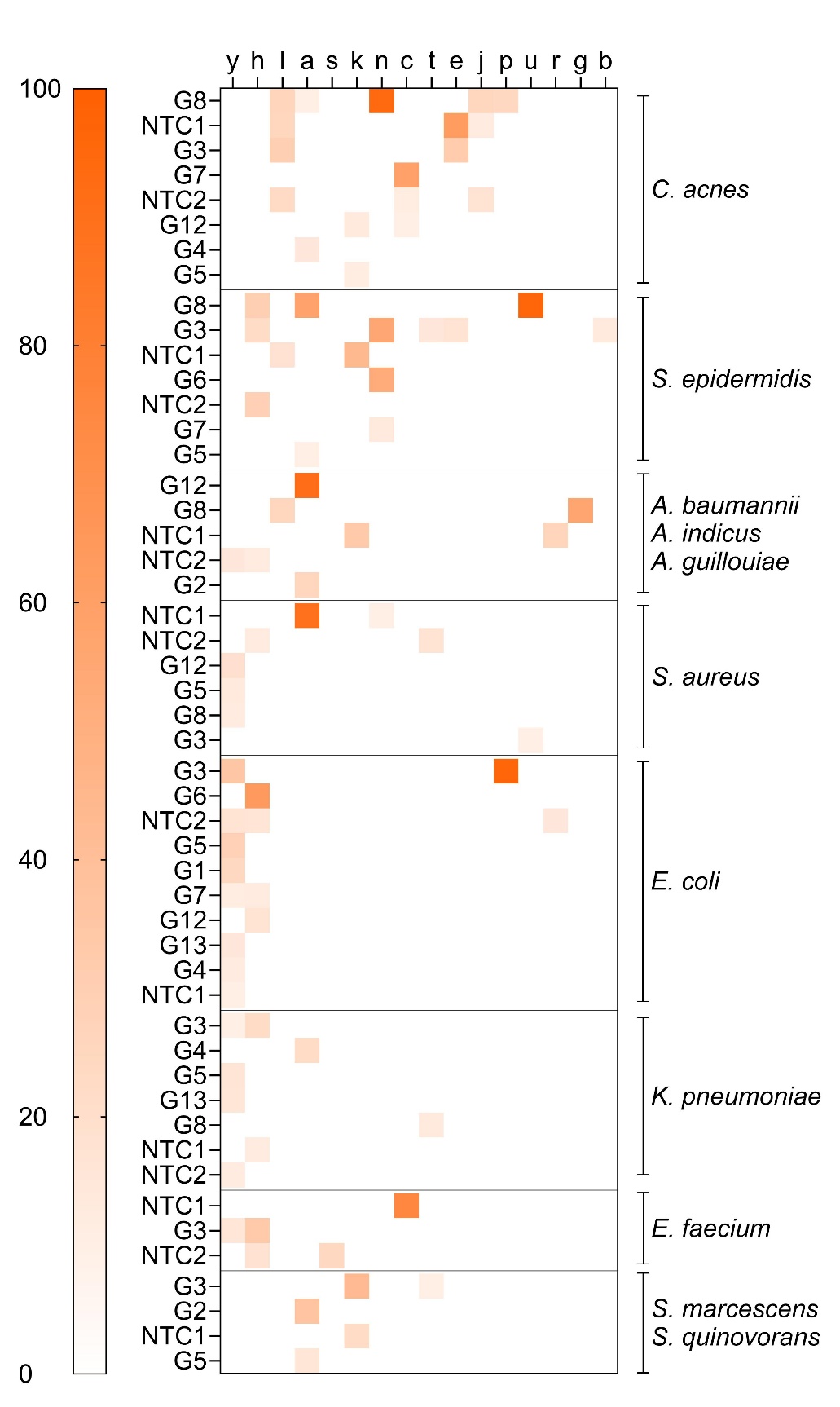


**Supplementary Figure S4.** The most abundant contaminant species detected using the GMS-16S pipeline. Data were reported if relative abundance was above 10 % and detected more than five times, by at least three separate laboratories (y-b).
